# Effect of ischemic preconditioning in skeletal muscle measured by functional magnetic resonance imaging and spectroscopy: a randomized crossover trial

**DOI:** 10.1186/1532-429X-13-32

**Published:** 2011-06-30

**Authors:** Martin Andreas, Albrecht I Schmid, Mohammad Keilani, Daniel Doberer, Johann Bartko, Richard Crevenna, Ewald Moser, Michael Wolzt

**Affiliations:** 1 Department of Clinical Pharmacology, Medical University of Vienna, Waehringer Guertel 18-20, A-1090 Vienna, Austria; 2 Center for Medical Physics and Biomedical Engineering, Medical University of Vienna, Waehringer Guertel 18-20, A-1090 Vienna, Austria; 3 MR Center of Excellence, Medical University of Vienna, Waehringer Guertel 18-20, A-1090 Vienna, Austria; 4 Department of Physical Medicine and Rehabilitation, Medical University of Vienna, Waehringer Guertel 18-20, A-1090 Vienna, Austria

## Abstract

**Background:**

Nuclear magnetic resonance (NMR) imaging and spectroscopy have been applied to assess skeletal muscle oxidative metabolism. Therefore, in-vivo NMR may enable the characterization of ischemia-reperfusion injury. The goal of this study was to evaluate whether NMR could detect the effects of ischemic preconditioning (IPC) in healthy subjects.

**Methods:**

Twenty-three participants were included in two randomized crossover protocols in which the effects of IPC were measured by NMR and muscle force assessments. Leg ischemia was administered for 20 minutes with or without a subsequent impaired reperfusion for 5 minutes (stenosis model). IPC was administered 4 or 48 hours prior to ischemia. Changes in ^31^phosphate NMR spectroscopy and blood oxygen level-dependent (BOLD) signals were recorded. 3-Tesla NMR data were compared to those obtained for isometric muscular strength.

**Results:**

The phosphocreatine (PCr) signal decreased robustly during ischemia and recovered rapidly during reperfusion. In contrast to PCr, the recovery of muscular strength was slow. During post-ischemic stenosis, PCr increased only slightly. The BOLD signal intensity decreased during ischemia, ischemic exercise and post-ischemic stenosis but increased during hyperemic reperfusion. IPC 4 hours prior to ischemia significantly increased the maximal PCr reperfusion signal and mitigated the peak BOLD signal during reperfusion.

**Conclusions:**

Ischemic preconditioning positively influenced muscle metabolism during reperfusion; this resulted in an increase in PCr production and higher oxygen consumption, thereby mitigating the peak BOLD signal. In addition, an impairment of energy replenishment during the low-flow reperfusion was detected in this model. Thus, functional NMR is capable of characterizing changes in reperfusion and in therapeutic interventions in vivo.

**Trial Registration:**

ClinicalTrials.gov: NCT00883467

## Background

The rapid reestablishment of perfusion may salvage ischemic tissue. However, reperfusion itself can result in additional cell damage, which is known as ischemia-reperfusion injury (IRI) [1,2]. Furthermore, a residual impairment of blood flow may limit functional recovery. The development of strategies aimed at mitigating IRI is hindered by a lack of experimental human models.

Ischemic preconditioning (IPC) is an established method to avoid IRI in different vascular beds [3-5]. The controlled repeated application of short periods of ischemia preceding a prolonged ischemic episode could also protect remote tissue against IRI [6]. The effects of IPC may be divided into an early phase of protection, which occurs during the first hours after IPC, and a late phase of protection, which is observed approximately 48 hours after IPC [7,8]. Previous data suggest that there is great variation in the amount of protection conferred by this mechanical intervention [6,7,9], and different protocols have been used for IRI attenuation in clinical studies.

We hypothesized that the effects of IPC and the reperfusion pattern in the ischemic lower leg muscle could be detected and further analyzed using high-field magnetic resonance. Therefore, 3-Tesla magnetic resonance spectroscopy (MRS) of ^31^P was applied in healthy subjects to quantify the levels of adenosine triphosphate (ATP), phosphocreatine (PCr) and inorganic phosphate (Pi) as well as intracellular pH. Blood oxygen level-dependent (BOLD) functional magnetic resonance imaging (fMRI) was also performed in these subjects.

Phosphorus MR spectroscopy is a validated technique to investigate ischemia and the effects of physical exercise [10-12]. The measurements obtained using this technique show a low variability in repeated testing and correlate closely with those determined for the whole body maximal oxygen uptake [13]. ^31^P spectroscopy provides a marker of mitochondrial function and the oxidative capacity of tissues [14].

BOLD fMRI has been previously applied to measure local blood oxygenation to evaluate cerebral blood flow as well as skeletal muscle and myocardial perfusion [15]. In patients with peripheral artery occlusive disease, BOLD imaging can characterize post-ischemic hyperemia [16] and the effects of percutaneous transarterial angioplasty [17]. This method is based on the differential effects of oxygenated and deoxygenated hemoglobin on the homogeneity and signal intensity of microscopic magnetic fields [18,19]. As additional outcome parameters, serum markers of muscle injury and muscle force were measured and compared to NMR results in the present study.

## Methods

The study was approved by the Ethics Committee of the Medical University of Vienna and conformed to the principles outlined in the Declaration of Helsinki, including current revisions and the Good Clinical Practice guidelines. The study protocol is registered at ClinicalTrials.gov (NCT00883467).

### Study design and population

This study comprised two different protocols. In the first protocol, 14 healthy male Caucasian subjects were subjected to NMR studies (age: 27 ± 7 years, body mass index: 22.4 ± 1.9 kg/m^2^). In the second protocol, 9 healthy male subjects (age: 27 ± 8 years, body mass index: 22.2 ± 1.3 kg/m^2^) were recruited to assess isometric muscle strength. Both protocols followed a randomized crossover design. After informed consent was obtained, all of the subjects underwent a complete health examination, including a physical examination, ECG and laboratory screening. The inclusion criteria were no history or signs of clinically relevant illness during the two weeks preceding the first day of the study and no contraindications for NMR scanning. The subjects were drug-free (including over-the-counter medications) for three weeks prior to the screening and until completion of the study. Four or eight study days with a washout interval of ≥6 days were scheduled for each participant according to a pre-defined protocol.

### Description of the NMR studies

The participants abstained from the consumption of alcohol and stimulating beverages containing xanthine derivatives for 12 hours before each trial period and were studied after an overnight fast. The participants also avoided heavy physical exercise for 3 days prior to the NMR and force measurements.

#### Preconditioning sequence

IPC was administered on two study days in each NMR protocol and on two study days together with the measurement of muscle force. In the NMR studies, the intervals between mechanical intervention and the initiation of measurements were 4 and 48 hours to detect the short- and long-term effects of IPC, respectively. Because long-term IPC had no effect on the NMR measurements in the first experiments, IPC was subsequently performed only 4 hours prior to the measurements obtained in the muscle force trial. IPC consisted of three 5-min periods of ischemia that were separated by two 10-min reperfusion intervals. For ischemia, a cuff that was inflated to 200 mm Hg was placed on the right thigh.

#### Administration of ischemia and low-flow reperfusion (stenosis)

Limb ischemia was administered to the right leg for 20 min using a thigh cuff that was inflated to suprasystolic pressure (200 mm Hg). The predetermined cuff pressure was achieved within four seconds of inflation using pressurized air. During the last two minutes of ischemia in the NMR protocol, the subjects performed plantar flexions at a half-maximal contraction force. These plantar flexions were carried out on an exercise rig every 4 seconds until exhaustion, as described previously [20]. Low flow reperfusion was induced by deflating the thigh cuff to 30 mm Hg below the systolic pressure for a total of 5 min following ischemia.

#### NMR recordings

**Figure 1:**
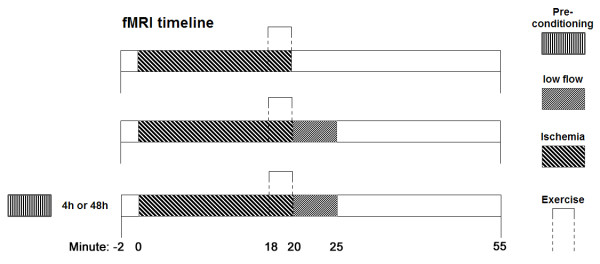
**Schema of NMR data acquisition**. Time course was equal for ^31^P spectroscopy and BOLD imaging. The first line shows the baseline day without low flow, the second line the day with low flow but without ischemic preconditioning (IPC) and the third line represents the two days with IPC.

NMR signals were acquired beginning at two min prior to ischemia until 30 min after release of the cuff (Figure 1). The measurements were performed using a 3T Tim Trio whole-body scanner (Siemens Medical Solutions, Erlangen, Germany). The right leg of the subject was fixed to a wooden exercise rig as described previously [10]. Changes in high-energy phosphate levels were recorded using a circular, double-resonant ^31^P/^1^H surface coil with a diameter of 105/95 mm (RAPID, Germany), which was positioned below the medial head of the right gastrocnemius muscle. A pulse-acquire procedure was used to collect the data. At the beginning of the experiment, the pulse was calibrated to maximize the signal yield. The dynamic scan lasted for approximately 50 min, during which a single acquisition was collected every 4 seconds. PCr and Pi resonances were quantified using AMARES [21] in jMRUI [22], pH was calculated from their frequency difference. Post-exercise post-ischemic PCr recovery was fitted to a single exponential equation plus a linear component to account for long-term instabilities in perl/PDL [23] using the Fit-Levmar module.

Blood oxygen level-dependent (BOLD) signals were calculated from fat-suppressed echo-planar images, recorded by a flexible coil wrapped around the subjects' calf (TR = 0.5 s, 90 deg. Flip angle, 5400 scans, TE= 44 ms, 128 pixels, 5 slices, 102 phase encodings, reconstruction to 128, 1.4 mm in-plane resolution, 5-mm-thick slices). Dicom images were exported and converted into the minc (http://www.bic.mni.mcgill.ca/ServicesSoftware/MINC) format for further processing. 100 images were blurred and averaged. This served as the reference to which all EPI were registered to correct for motion. They were resampled in the slice direction because the registration was done in three dimensions to correct also for through plane motion. A full 12 parameter linear registration the minctracc utility was used to calculate the transformation. To improve signal to noise ratio (SNR) and to minimize computation time, the parameters were calculated for five averaged scans. After image registration, manually drawn regions of interest (ROI) covering the soleus, gastrocnemius and tibialis anterior muscle were extracted. Large vessels were filtered based on the characteristic hyperintense signal which disappears during ischemia. Afterwards the signal was summed to achieve a time course for each individual muscle. This was then characterized by taking values at predefined points.

### Assessment of isometric muscular strength

Four trial days were scheduled for each participant. On two days, ischemia-reperfusion was administered both with and without postischemic stenosis. On the other two days, IPC was performed four hours prior to ischemia both with and without post-ischemic stenosis. The cuff pressure reduction to 30 mm Hg below systolic pressure caused post-ischemic stenosis. Muscular strength was measured prior to ischemia, during the last 2 min of ischemia and every 5 min after reperfusion for a total of 20 minutes. The Biodex 3 dynamometer (Biodex Medical Systems, Shirley, New York, USA) was used to quantify the isometric plantar flexion/dorsiflexion strength of the right leg ankle muscles, according to the specifications of the manufacturer [24-26].

The participants performed three sets of maximal voluntary isometric plantar flexion/dorsiflexion contractions (ankle angle: 15° flexion, knee flexion: 20-30°), which were maintained for five seconds each. The maximal isometric force was measured and normalized to body weight. During ischemia, only plantar flexion strength was measured, and the results corresponded to the muscular work performed in the exercise rig.

### Laboratory analysis

Venous blood was collected during the NMR studies. Blood was collected to measure the levels of creatine kinase, lactate dehydrogenase, free hemoglobin and C-reactive protein before ischemia and at 5 min, 15 min, and 24 hours after reperfusion.

### Statistical Analysis

The data sets were analyzed descriptively, and the results are presented as the mean ± SD or the median (quartiles) for parametric and non-parametric data, respectively. To compare the outcome parameters among the groups, analysis of variance or the Kruskal-Wallis test was used to evaluate parametric and non-parametric data, respectively. P-values less than 0.05 were considered significant. All statistical analyses were performed using SPSS V17 for Macintosh (SPSS Inc., Chicago, Illinois, USA).

## Results

Four participants completed both NMR protocols, four completed ^31^P MRS studies and six completed BOLD fMRI studies. There were no changes in blood pressure or in the concentrations of circulating creatine kinase, free hemoglobin or C-reactive protein across the study periods (additional file 1 + 2). IPC 48 hours prior to ischemia decreased lactate dehydrogenase serum concentrations from 197 ± 18 U/l to 156 ± 16 U/l (p < 0.006, *n*=8) before the application of ischemia (additional file 2).

### ^31^P MRS

**Figure 2:**
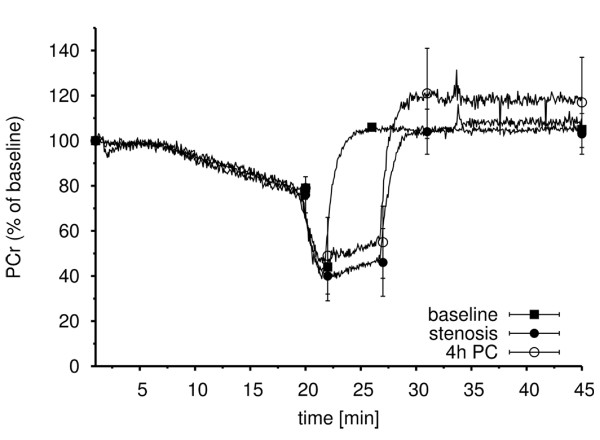
**^31^P time course**. Time course of phosphocreatine (PCr) at baseline, during ischemia and reperfusion, and in the presence of post-ischemic stenosis with or without mechanical preconditioning four hours before ischemia. The data represent the means ± SEM (*n*=8).

The PCr signal decreased substantially during ischemia and exercise to 44 ± 13% of the baseline value (Table 1, Figure 2). Following complete reperfusion, the PCr signal recovered quickly, the average time constants are reported in Table 1. Normalized levels were observed within 230 ± 102 seconds. In contrast, the PCr signal increased modestly during the 5-min post-ischemic stenosis period from trough values of 40 ± 11% to 46 ± 15% of the baseline value. A rapid recovery of the PCr signal was again observed within 243 ± 83 seconds after full reperfusion (Table 1, Figure 2). Pi increased during ischemia and exercise (Table 1). The effect of reperfusion was similar with or without preceding stenosis. Reperfusion significantly reduced Pi from the baseline NMR signal (p < 0.001). PCr signals normalized at the end of the observation period. pH values increased slowly during ischemic rest and dropped rapidly during exercise and initial recovery (Table 2). They slowly returned to baseline values during further recovery. During stenosis, pH decreased slightly.

**Table 1:** Phosphocreatine and Pi MRS signals

Phosphocreatine (% of baseline)	Ischemia and exercise	Post-ischemic stenosis	Post-ischemic stenosis and 4 h IPC	Post-ischemic stenosis and 48 h IPC
*Number of subjects*	*(n = 7)*	*(n = 8)*	*(n = 8)*	*(n = 8)*
Ischemia	79 ± 3*	76 ± 8*	79 ± 5	76 ± 3*
Ischemic exercise	44 ± 13*	40 ± 11*	49 ± 17*	39 ± 12*
Post-ischemic stenosis	-	46 ± 15*	55 ± 16*	45 ± 17*
Reperfusion	106 ± 5	104 ± 10	121 ± 20†	109 ± 9
End	105 ± 3	103 ± 9	117 ± 20	108 ± 13

PCr recovery time-constant (s)	46 ± 20	49 ± 17	43 ± 15	47 ± 17

Inorganic phosphate (% of baseline)	Ischemia and exercise	Post-ischemic stenosis	Post-ischemic stenosis and 4 h IPC	Post-ischemic stenosis and 48 h IPC
*Number of subjects*	*(n = 7)*	*(n = 8)*	*(n = 8)*	*(n = 8)*

Ischemia	334 ± 44*	305 ± 62*	321 ± 89*	310 ± 48
Ischemic exercise	731 ± 183*	657 ± 203*	681 ± 193*	654 ± 281*
Post-ischemic stenosis	-	558 ± 229*	578 ± 165*	532 ± 260*
Reperfusion	51 ± 21	37 ± 15	58 ± 24	55 ± 26
End	110 ± 11	90 ± 84	116 ± 34	92 ± 25

Mechanical preconditioning (IPC) was applied 4 h or 48 h before ischemia.

Data represent the means ± SD. The baseline is defined as the NMR signal prior to ischemia. IPC: ischemic preconditioning, * p < 0.05 versus baseline, † p = 0.05 vs. day of post-ischemic stenosis

**Table 2:** pH MRS signals

pH	Ischemia and exercise	Post-ischemic stenosis	Post-ischemic stenosis and 4 h IPC	Post-ischemic stenosis and 48 h IPC
*Number of subjects*	*(n = 7)*	*(n = 8)*	*(n = 8)*	*(n = 8)*
Baseline	7.03 ± 0.02	7.05 ± 0.03	7.05 ± 0.03	7.07 ± 0.05
Ischemia	7.08 ± 0.02*	7.10 ± 0.04*	7.09 ± 0.02*	7.09 ± 0.02
Ischemic exercise	6.93 ± 0.14	6.92 ± 0.14	6.92 ± 0.17	6.98 ± 0.14
Post-ischemic stenosis	-	6.88 ± 0.12	6.91 ± 0.15	6.96 ± 0.14
Reperfusion	6.85 ± 0.18	6.86 ± 0.12*	6.89 ± 0.17	6.90 ± 0.17
End	7.04 ± 0.02	7.10 ± 0.04	7.07 ± 0.08	7.08 ± 0.02

Mechanical preconditioning (IPC) was applied 4 h or 48 h before ischemia.

Data represent the means ± SD. The baseline is defined as the NMR signal prior to ischemia. IPC: ischemic preconditioning, * p < 0.05 versus baseline

IPC four hours prior to ischemia/exercise and post-ischemic stenosis significantly increased the maximal PCr reperfusion signal intensity compared to ischemia/exercise and post-ischemic stenosis alone (Table 1, Figure 2), but had no influence on the speed of recovery. IPC 48 hours before ischemia had no effect on the time course of PCr. IPC had no significant impact on pH or Pi. During the measurements, no changes in ATP were observed.

### BOLD fMRI

**Figure 3:**
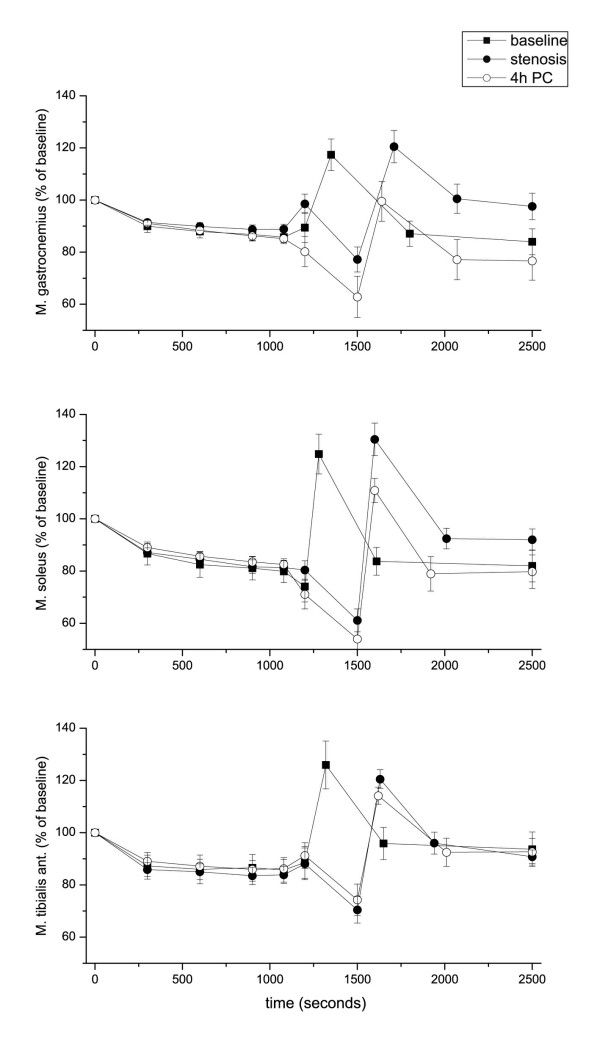
**BOLD time course**. Time course of the BOLD signal in the gastrocnemius, soleus, and tibialis anterior muscles at baseline, during ischemia and reperfusion, and in the presence of post-ischemic stenosis with or without mechanical preconditioning four hours prior to ischemia. The data represent the means ± SEM (*n*=8).

**Figure 4:**
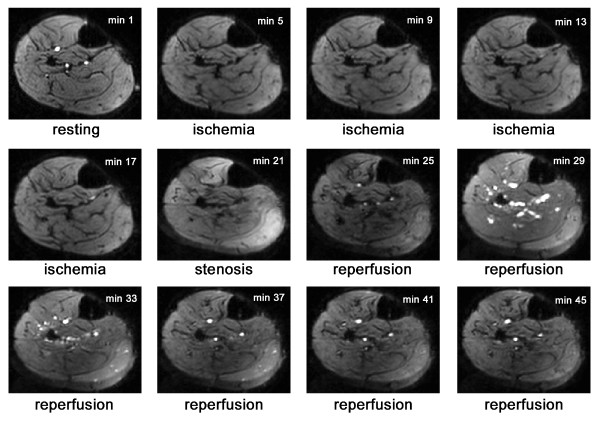
**Echoplanar images for the BOLD signal analysis**. The images are representative of the BOLD source data. An image for every 4^th ^minute of data collection is shown.

Ischemia rapidly decreased the BOLD signal intensity within the first 100 seconds of cuff occlusion. The BOLD signals were stable at 88 ± 6%, 86 ± 8%, 84 ± 9% and 84 ± 9% of baseline after 5, 10, 15 and 18 min of ischemia, respectively (Figures 3 and 4). Post-ischemic stenosis produced an additional decline of the BOLD signal to 70 ± 17% of the baseline value. During complete reperfusion, the maximum signal intensity was 124 ± 15% and 123 ± 16% of the baseline value with and without post-ischemic stenosis, respectively.

IPC 4 hours prior to ischemia did not affect the BOLD signal decline during ischemia, but it mitigated the peak BOLD signal following post-ischemic stenosis during reperfusion to 108 ± 13% of the baseline value (p = 0.029 vs. no IPC). This effect of IPC was evident in the gastrocnemius and soleus muscles, which were exhausted by anaerobic exercise, but it was not observed in the tibialis anterior muscle (Table 3, Figures 3 and 4). IPC 48 hours prior to ischemia had no effect on the peak BOLD signal after the post-ischemic stenosis.

**Table 3:** Maximum BOLD muscle signal (% of baseline) after IRI and stenosis with or without IPC.

	no IPC (*n *= 8)	4 h IPC (*n *= 8)	48 h IPC (*n *= 9)
All muscles	124 ± 15	108 ± 13*	123 ± 11
Tibialis anterior muscle	121 ± 12	113 ± 10	128 ± 19
Soleus muscle	130 ± 20	109 ± 14*	129 ± 19
Gastrocnemius muscle	123 ± 18	101 ± 24*	114 ± 8

Data represent the means ± SD. IPC: ischemic preconditioning, *p < 0.05 vs. no IPC

### Isometric muscle strength measurement

One participant withdrew his consent prior to the first day of the study, and the measurements for two subjects were unavailable for analysis on two study days for technical reasons.

**Figure 5:**
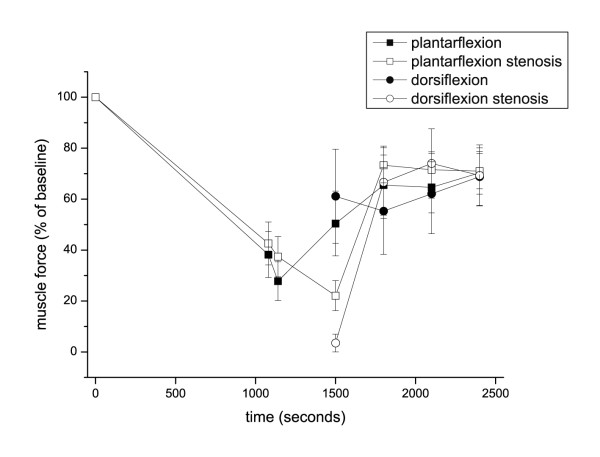
**Muscle strength time course**. Ankle plantar flexion and dorsiflexion strength before and during ischemia and after reperfusion with or without post-ischemic stenosis. The data represent the means ± SEM (*n*=7).

The maximum ankle plantar flexion and dorsiflexion strengths were 129 ± 48 Nm and 48 ± 14 Nm at baseline, respectively. During ischemia, plantar flexion strength was reduced to 28 ± 17% of the baseline value. Post-ischemic stenosis for five min further decreased plantar flexion strength to 22 ± 16% of baseline (Figure 5). After 5, 10 and 15 min of complete cuff release, muscular strength recovered to 66 ± 31%, 65 ± 24% and 70 ± 21% of the baseline value in subjects without post-ischemic stenosis and to 73 ± 18%, 71 ± 19% and 71 ± 18% of the baseline value in subjects with post-ischemic stenosis (p = ns).

Plantar dorsiflexion strength was also reduced in response to ischemia. After the post-ischemic stenosis, muscle strength was 4 ± 9% of the baseline value (p = 0.007 vs. complete reperfusion). After 5, 10 and 15 min of complete cuff release, dorsiflexion strength recovered to 55 ± 50%, 62 ± 39% and 69 ± 28% of the baseline value in subjects without post-ischemic stenosis and to 67 ± 38%, 74 ± 36% and 69 ± 32% of the baseline value in subjects with post-ischemic stenosis (p = ns).

IPC 4 hours before ischemia had no effect on the time course of ankle plantar flexion or on dorsiflexion strength recovery.

## Discussion

Multi-modal *in vivo *NMR in IRI enables the evaluation of mitochondrial oxidative metabolism, muscle metabolic changes and changes in perfusion. The additional measurement of functional skeletal muscle deficits allows for the interpretation of NMR data and their relationship with force recovery.

### Preconditioning effect on NMR signals during reperfusion

The results of the present study demonstrate the influence of IPC on PCr and the BOLD signal. Whereas IPC was unable to prevent PCr depletion during ischemia or post-ischemic stenosis, peak concentrations of PCr after reperfusion were substantially higher after IPC compared to the control group. This effect was only observed when IPC was applied 4 hours prior to ischemia. A mitigation of reperfusion-induced increases in the BOLD signal amplitude was consistently recorded by BOLD imaging in subjects who were exposed to IPC 4 hours before ischemia. IPC 48 hours before ischemia did not affect the PCr and BOLD signal intensity.

### ^31^P MRS signal changes

Phosphorus MR spectroscopy was applied to assess muscle metabolism in different populations, conditions and diseases. The simultaneous measurement of ATP, PCr and Pi, and consequently pH, provides insight into the energy status of the tissue and its capacity to undergo oxidative phosphorylation. The effect of IPC on pH was not significant. The PCr time course was the most useful analysis for elucidating the effects of IPC on IRI. This is the first study to demonstrate an effect of IPC using ^31^P MRS in human skeletal muscle. However, Miyamae et al. have previously described the effects of IPC on ^31^P spectroscopy in the porcine heart [27], in which, comparable to the present findings, a pronounced PCr overshoot was induced by IPC.

IRI induces cell death primarily via impaired and altered mitochondrial functions. Rapid ATP restoration is crucial for the re-establishment of mitochondrial homeostasis and the prevention of additional cell damage [28]. RNA and protein synthesis are particularly sensitive to ATP/ADP ratio, and PCr overshoot was reported in conjunction with increased ATP/ADP ratio [29]. PCr overshoot was consistently related to IPC in a repeated measurements ANOVA and was not limited to a distinct subset of trial participants [30]. PCr overshoot without IPC was reported to be accompanied by acidification as well as alterations in ATP/ADP ratio and Pi [30]. In the present study, however, PCr overshoot was measured without any of these other effects after IPC. The underlying mechanism of the protective effect of IPC may be a prolonged and increased parallel activation of oxidative phosphorylation independent of pH or ADP levels, leading to increased repair capacity of skeletal muscle cells [31]. We therefore conclude that IPC prepares cells to stimulate cellular metabolism during recovery.

The relatively short period of ischemia employed in this study did not result in persistent cellular or mitochondrial damage and reduced muscular ATP content. No measureable harm was done to the muscle according to pre- and postprocedural serum creatine-kinase concentrations. IPC did not have any significant influence on PCr or pH during resting ischemia and exercise. The protective mechanisms of preconditioning are active during the reperfusion period and not during ischemia, which is in good agreement with the concepts of ischemic preconditioning to act against reperfusion injury [7]. Therefore, PCr decrease during insult may not be appropriate to detect IPC effects. No significant difference in creatine rephosphorylation rates could be observed. This is in agreement with unaltered pH and ATP/ADP ratio. We conclude that IPC is a protective mechanism by activating cellular metabolism to be eventually prepared for excessive repair tasks.

### BOLD signal changes

The initial rapid decrease in the BOLD signal during ischemia is caused by hemoglobin deoxygenation [32,33]. During reperfusion, the signal increase is attributed to an elevated delivery of oxygenated hemoglobin and vasodilatation [34]. In addition, the BOLD signal is influenced by the anatomic and vascular muscle structure and the (de)oxygenation of myoglobin [35-37]. The effects of post-ischemic stenosis on venous return may explain the additional decrease in the BOLD signal during impaired reperfusion [34]. The venous system fills continuously due to a slow arterial inflow, which increases the amount of deoxygenated hemoglobin and reduces the BOLD signal.

IPC significantly reduced the peak BOLD signal during hyperemia. The protection provided by IPC was most pronounced in the gastrocnemius muscle, which is a fast-twitch glycolytic muscle and is the main contributor to force in repeated plantar flexions [35]. Therefore, this finding may be explained by a greater exhaustion of this muscle or by a muscle type-specific signal pattern [32]. As previously described, the highest peak BOLD signal during reperfusion is observed in the soleus muscle [38]. The soleus muscle is a slow-twitch muscle that is rich in capillaries and myoglobin, which likely account for the pronounced BOLD effect [39].

Flow-mediated hyperemia is a test of forearm resistance and conduit vasculature that is commonly applied to assess endothelial function. IPC has been previously shown to preserve forearm endothelial function after 20 min of ischemia [40]. IRI leads to a decrease in hyperemic flow-mediated dilatation that can be overcome by remote ischemic preconditioning. In contrast, the present data showed a less pronounced hyperemic peak in the muscular maximal BOLD signal (Table 3). Thus, muscular BOLD-based imaging likely reveals far more complex information than solely flow-mediated dilatation because it integrates the effects of the diameter and recruiting of capillaries, the myoglobin saturation status, oxygen consumption and muscle-specific patterns. When muscle exercise is performed on top of ischemia, the interpretation of BOLD signals becomes more complex and thorough interpretation would require simultaneous monitoring with Doppler flow measurement, arterial spin labeling or near infrared spectroscopy.

### Multimodal ischemia-reperfusion assessments explain the BOLD signal reduction

An increase in PCr formation was observed during reperfusion four hours after IPC, which indicated that energy metabolism was improved during the post-ischemic period. This counter-regulatory PCr production was most likely due to an increase in oxidative phosphorylation in the reperfused tissue. PCr production depends on the ATP/ADP ratio and the mitochondrial creatine kinase. It is produced by oxidative phosphorylation [41]. The opposite effect (reduced PCr depletion during the post-ischemic period due to IPC) appears to be unlikely because PCr levels in the presence of IPC are higher than the pre-ischemic values. Thus, we suspect that there is an increased oxidative phosphorylation in reperfused tissue after IPC and IRI. This increases the demand for oxygen in preconditioned tissue after IRI and the formation of an oxygen gradient. Therefore, a reduced BOLD signal during the reperfusion period four hours after IPC may be readily explained by an increased oxygen demand in muscle cells and therefore a reduced ratio of oxygenated versus deoxygenated hemoglobin in comparison to non-IPC tissue. This hypothesis, however, remains to be proven.

### Systemic laboratory effects of IRI

IPC 48 hours prior to ischemia decreased the levels of lactate dehydrogenase, as described previously [42]. However, a reduction of lactate dehydrogenase was observed before the onset of ischemia. Because the levels of lactate dehydrogenase were within the normal range throughout the observation period, this result may be insignificant.

### Impaired reperfusion inhibits PCr recovery

Impaired reperfusion had a potent influence on the recovery of PCr in healthy subjects. The decline of PCr during ischemia is consistent with previous data [10,12]. However, low-flow reperfusion prevented the recovery of PCr and was paralleled by a further reduction of the BOLD signal and a decrease in muscular strength. The levels of ATP were stable during the entire process. This result may be explained by the increased ATP production via glycolysis in the cytosol, supported by decreasing pH values, which is induced by the preceding ischemia and ischemic contractions [43]. Because it is driven by mitochondrial creatine kinase, PCr synthesis depends on mitochondrial ATP generation and is independent of cytosolic ATP production. Therefore, our data indicate that there is an insufficient level of oxidative phosphorylation during low-flow reperfusion and that ATP is stabilized via glycolytic ATP generation, which is comparable to that observed during hypoxic perfusion [44].

Skeletal muscle PCr was restored far more rapidly after full reperfusion in comparison to muscular strength. In addition, muscular strength further decreased during stenotic reperfusion despite a stable ATP concentration. This seemingly unexpected finding may be readily explained by the data reported by Lanza et al., who state that the reduction of ATP consumption during ischemic contractions is due to increased muscular fatigue [44]. The complex mechanisms behind the process of muscular fatigue have not been fully understood yet. A substantial component may be related to changes in intracellular pH.

Therefore, muscular strength may not be directly affected by PCr or ATP synthesis, thus explaining different recovery times. Although the subjects exhibited some variability in muscular strength and in their reactions to the ischemic stimulus, a prolonged force impairment in the calf muscle was maintained. Therefore, our data highlight the importance of full and rapid reperfusion (as opposed to low-flow reperfusion) for the rapid recovery of muscle force.

### Limitations

Only male and healthy subjects were studied in this trial. Further studies are required to assess these parameters in various disease states and take gender aspects into account. However, the present data may serve as a reference in further clinical trials. In addition, the sample size in the present study was relatively small. Ischemia-reperfusion tests cause pain and tissue injury, and ethical constraints limit the duration of the experimental ischemia. Prolonged ischemia may involve pathophysiological mechanisms in addition to those that occur after a short-term flow impairment.

It must be noted that although similar findings have also been reported for cardiac muscle, the present results cannot be extrapolated to cardiac muscle or to other tissues.

## Conclusions

Combined high-field ^31^P MRS and BOLD imaging are suitable for the evaluation of ischemia and reperfusion mechanisms in skeletal muscle and may be used to test therapeutic strategies in humans.

## Competing interests

The authors declare that they have no competing interests.

## Authors' contributions

All authors fulfill the criteria for authorship. MA, DD and MW designed and drafted the interventional protocol, AS and EM developed and applied the fMRI protocols. MK and RC performed and drafted the measurement of muscular strength. JB analyzed and evaluated the laboratory data. MA and MW wrote the final draft. All authors have read and approved submission of the final draft.

## Supplementary Material

Additional file 1 **Blood pressure and C-reactive protein**. This table presents a descriptive statistic of blood pressure and C-reactive protein values prior to and after ischemia for every study day. Click here for file

Additional file 2 **Free hemoglobin, lactate dehydrogenase and creatine kinase**. This table presents a descriptive statistic of free hemoglobin, lactate dehydrogenase and creatine kinase values prior to and after ischemia for every study day. Click here for file
