# Supplementary material for: Effect of ischemic preconditioning in skeletal muscle measured by functional magnetic resonance imaging and spectroscopy: a randomized crossover trial
Source: J Cardiovasc Magn Reson. 2011 Jun 30;13(1):32. doi: 10.1186/1532-429X-13-32 (PMC3143996; doi:10.1186/1532-429X-13-32)
Supplement: Additional file 1 — Blood pressure and C-reactive protein. This table presents a descriptive statistic of blood pressure and C-reactive protein values prior to and after ischemia for every study day. [file 1532-429X-13-32-S1.DOC]

**Additional file 1**

**Table 1: Blood pressure and C-reactive protein**

|  | Ischemia and exercise | Post-ischemic stenosis | Post-ischemic stenosis and 4h IPC | Post-ischemic stenosis and 48h IPC |
| --- | --- | --- | --- | --- |
| **Systolic blood pressure** |  |  |  |  |
| 48 hours prior ischemia | - | - | - | 116±11 |
| 4 hours prior ischemia | - | - | 114±8 | - |
| Prior to ischemia | 114±7 | 114±9 | 114±8 | 111±7 |
| After ischemia | 113±7 | 114±10 | 113±7 | 112±8 |
| **Diastolic blood pressure** |  |  |  |  |
| 48 hours prior ischemia | - | - | - | 70±12 |
| 4 hours prior ischemia | - | - | 73±2 | - |
| Prior to ischemia | 78±10 | 79±7 | 78±7 | 76±6 |
| After ischemia | 80±4 | 79±8 | 80±5 | 77±7 |
| **C-reactive protein** |  |  |  |  |
| 48 hours prior ischemia | - | - | - | 0.05±0.03 |
| 4 hours prior ischemia | - | - | 0.23±0.55 | - |
| Prior to ischemia | 0.08±0.1 | 0.1±0.2 | 0.21±0.53 | 0.04±0.02 |
| After ischemia | 0.08±0.1 | 0.1±0.2 | 0.18±0.42 | 0.04±0.01 |
| 24 hours after ischemia | 0.09±0.1 | 0.09±0.2 | 0.18±0.42 | 0.03±0.00 |
