# Supplementary material for: Effect of ischemic preconditioning in skeletal muscle measured by functional magnetic resonance imaging and spectroscopy: a randomized crossover trial
Source: J Cardiovasc Magn Reson. 2011 Jun 30;13(1):32. doi: 10.1186/1532-429X-13-32 (PMC3143996; doi:10.1186/1532-429X-13-32)
Supplement: Additional file 2 — Free hemoglobin, lactate dehydrogenase and creatine kinase. This table presents a descriptive statistic of free hemoglobin, lactate dehydrogenase and creatine kinase values prior to and after ischemia for every study day. [file 1532-429X-13-32-S2.DOC]

**Additional file 2**

**Table 2: Free hemoglobin, lactate dehydrogenase and creatine kinase**

|  | Ischemia and exercise | Post-ischemic stenosis | Post-ischemic stenosis and 4h IPC | Post-ischemic stenosis and 48h IPC |
| --- | --- | --- | --- | --- |
| **Free hemoglobin** |  |  |  |  |
| 48 hours prior ischemia | - | - | - | 6.4±5.8 |
| 4 hours prior ischemia | - | - | 8.0±6.5 | - |
| Prior to ischemia | 9.7±6.2 | 15.4±6.8 | 14.9±8.4 | 20.1±39.2 |
| After ischemia | 4.9±2.2 | 3.7±2.2 | 4.9±2.4 | 3.3±2.0 |
| 24 hours after ischemia | 3.7±2.4 | 7.2±7.8 | 5.9±4.8 | 14.2±13.9 |
| **Lactate dehydrogenase** |  |  |  |  |
| 48 hours prior ischemia | - | - | - | 157±16 |
| 4 hours prior ischemia | - | - | 167±19 | - |
| Prior to ischemia | 178±19 | 197±18* | 189±26 | 156±16* |
| After ischemia | 156±18 | 153±15 | 154±16 | 141±12 |
| 24 hours after ischemia | 156±19 | 170±39 | 160±26 | 167±23 |
| **Creatine kinase** |  |  |  |  |
| 48 hours prior ischemia | - | - | - | 151±77 |
| 4 hours prior ischemia | - | - | 124±38 | - |
| Prior to ischemia | 165±81 | 183±86 | 145±52 | 159±60 |
| After ischemia | 150±73 | 166±74 | 131±49 | 147±51 |
| 24 hours after ischemia | 169±91 | 205±101 | 145±57 | 172±95 |

* p=0.006
